# Models in complimentary medicine

**DOI:** 10.4103/0973-6131.78169

**Published:** 2011

**Authors:** Thaiyar M Srinivasan

**Affiliations:** Swami Vivekananda Yoga Anusandhana Samsthana (A Yoga University), No.9, Appajappa Agrahara, Chamarajpet, Bangalore - 560 018, India. E-mail: editor@ijoy.org.in

Although a great deal of research in alternative and complimentary medicine has been reported in the recent past, two questions remain unresolved. The first is with regard to the modality of activity that is related to the models that these therapies work with. The second is the lack of dose-response relation that is important in biochemical medicine. Both these are of importance in drug research and in ‘targeted’ physiology. The biochemical model is very strong in providing a basis for the activity of any drug under investigation. The drugs are also thought to be very specific in their activity and the dose-response is well documented. This is not to say that *all* drug-induced interactions are investigated and reported; often, the side effects are more profound than the effect, due to intended activity. Thus, it is reported that iatrogenesis — which are complications due to prescription drugs and procedures in hospitals — is perhaps the largest killer in the USA.[1]

Some models are based on ancient sciences such as Yoga, Ayurveda, and Acupuncture. A few seem to think that the present understanding in Physics could rescue the unknown through a known paradigm. This is bound to create difficulties, as the effect defies the present knowledge-base in the conventional sciences. Hence, invoking and conjoining ancient and modern terminologies only provided further ammunition to the uninitiated. Further books and articles have been written on the quantum nature of healing and cure, instant meditation techniques, and so on. Some paranormal powers are also attributed to people who seem to defy normal ways of handling information. There are books written by the psychically enabled, to provide what seems to be a model that provides more occasion to throw a spanner in the works by the psychically disabled!

There is some good news in the confusing scenario of model building. There are models based on the new physics of biology. Here, some important books have emerged by concerned physicists who have examined the phenomenon of healing from a different perspective and offered what could be new and innovative methods of understanding natural events in our lives. In Patanjali’s Yoga Sutras, many siddhis (or extraordinary powers) are enumerated that need some explanation. Perhaps these models could be useful in explaining some of the phenomena observed in psychics. We shall briefly review two models that are of interest in this connection.

## SOLITON PROPAGATION

A soliton is a self-reinforcing solitary wave that maintains its shape while it travels at constant speed. John Scott Russell in the mid-nineteenth century observed a solitary wave in a water canal in Scotland, which did not disappear as a normal wave, but persisted for many miles.[2] Solitons are caused by a cancellation of nonlinear and dispersive effects in the medium. (Dispersion is a property of many transmission systems where the wave velocity changes as the frequency changes). Solitons arise as a solution of a class of nonlinear dispersive partial differential equations, describing the physical systems.

The soliton model is also invoked in neurosciences that attempt to explain how signals are conducted through the neuronal network. The signals seem to travel along the membrane of a cell in the form of acoustic soliton pulses. This is an alternative to the widely accepted Hodgkin-Huxley model, which proposes that signals travel as action potentials. In the H-H model, the membrane channels open and allow sodium ions to rush into the cell, thereby leading to the opening of other nearby ion channels and thus propagating the signal in an essentially electrical manner. If one takes the thermodynamic properties of the nerve under propagation, it is probable that the soliton model is a more likely one.[3]

Healing ‘energy’ can work — so it seems — over long distances; in other words, this energy does not decay and disappear with distance. It seems to have the characteristics of soliton wave propagation. As neurons seem to transmit soliton waves, it is possible that these waves could couple to other systems in the body such as communication in the brain and the spinal cord. Radiation of these waves over large distances could then be possible, making prayers and other related healing phenomena to be explained through the soliton model. This model has not been investigated in detail so far.

Another interesting model is the so-called Biofield Hypothesis.[4] There are extemely weak electromagnetic fields inside the organisms that are involved in growth, repair, and healing, which happens all the time within the body. Furthermore, any intervention could be a structural, biochemical or regulatory one. An example of a structural change is the use of surgical techniques for removing an obstruction or unwanted growth. Biochemical interventions are the well-known biomedical drugs and naturally occuring herbs. Lastly, the regulatory ones are those related to genetic and weak electromagnetic information that control all aspects of cell dynamics. The biofield hypothesis is thought to be useful in understanding the scientific basis of energy medicine, which includes such diverse practices as homeopathy, acupuncture, yoga, and various healing methods. Let us look at the biofield hypothesis in some detail.

The biofield is defined as a dynamic electromagnetic (EM) field that is endogenous (occurring within the body itself) to an organism. It is involved in self-regulation and control. Each cell, tissue, and organ can be considered as generating and being influenced by this complex electromagnetic field. The pattern of EM waves that emerge can be highly complex maintaining order, and hence, the health of the entire organism. One might ask if there is any support available for this hypothesis. Many EM therapies used in rehabilitation (such as Transcutaneous Electrical Nerve Stimulation (TENS) for pain control) or in support of a failing organ (cardiac pacemakers) work on the principle of supplementing the biofield through external sources.

Limb regeneration studies provide yet another evidence for biofield hypothesis. Herein minute voltages seem to correct the biofield and growth of new tissues is possible.[5,6] There are many other therapies, including acupuncture, homeopathy, and yoga that could induce the biofield for homeostatic mechanisms. Furthermore, any organism or a living entity seems to possess an innate biofield that maintains the health of the organism. Health is simply the maintenance of the correct field characteristics and disease is the disruption of the field, either locally or globally, within the organism. It is possible that we can generate equations that can connect the biofield with the biochemical organization of the organism. In other words, it is highly probable that the biofield is the fundamental organizing principle that provides a living matrix for the living system.[6]

The two models presented above constitute only the tip of the proverbial iceberg. There are many models of healing that are based on quantum physics and quantum chemistry. However, the above models are sustained by many observations and it is interesting how these models can be expanded to provide a base for the various therapies available today.
